# Recurrent Cerebral Venous Sinus Thrombosis Occurred in an Acute Lymphoblastic Leukemia Child with Mutated Lipoprotein Lipase Gene during Asparaginase Therapy

**DOI:** 10.1055/s-0044-1788043

**Published:** 2024-07-05

**Authors:** Shiyuan Wang, Jun Li, Ying Li, Xiaoming Liu, Lixian Chang, Beibei Zhao, Li Zhang, Yao Zou, Min Ruan, Xiaofan Zhu

**Affiliations:** 1 State Key Laboratory of Experimental Hematology, Pediatric Hematology and Oncology Center, National Clinical Research Center for Blood Diseases, Haihe Laboratory of Cell Ecosystem, Institute of Hematology and Blood Diseases Hospital, Chinese Academy of Medical Sciences and Peking Union Medical College, Tianjin, China; 2 Tianjin Institutes of Health Science, Tianjin, China; 3 State Key Laboratory of Experimental Hematology, Department of Radiology, National Clinical Research Center for Blood Diseases, Haihe Laboratory of Cell Ecosystem, Institute of Hematology & Blood Diseases Hospital, Chinese Academy of Medical Sciences & Peking Union Medical College, Tianjin, China

**Keywords:** acute lymphoblastic leukemia, gene mutation, L-Asparaginase, cerebral venous sinus thrombosis

## Abstract

Cerebral venous sinus thrombosis (CVST) and hyperlipidemia are severe complications of L-Asparaginase (L-Asp) during the treatment of B-cell acute lymphoblastic leukemia (B-ALL). Herein, we reported a 9-year-old B-ALL boy who underwent abnormal hypertriglyceridemia and CVST presenting as seizures and disturbance of consciousness twice during the induction therapy. Fortunately, he survived treatment with anticoagulant and lipid-lowering therapy. No thrombophilia-related gene mutation was detected, but a heterozygous mutation in lipoprotein lipase (LPL) gene was identified. His neurological symptoms were managed with short-term anticoagulant therapy and long-term lipid-lowering therapy. This case illustrated the manifestation and potential pathogenesis of CVST and highlighted the essentiality of screening baseline lipid profile and dyslipidemia- and thrombophilia-related gene mutation.

## Introduction

Acute lymphoblastic leukemia (ALL) is the most prevalent childhood malignancy and constitutes approximately 25% of cancer diagnoses among children, 1 2 and the long-term overall survival and event-free survival rate of pediatric ALL has exceeded 90 and 85%. 1 3 L-Asparaginase (L-Asp) is a basic component of chemotherapy in pediatric ALL but comes with numerous side effects, including affecting lipid metabolism and inciting thrombosis. 4 Cerebral venous sinus thrombosis (CVST) is a rare complication during the treatment of ALL, presenting as seizures, hemiplegia, severe headaches, and coma.

## Case Report

A 9-year-old boy (weight: 35 kg, height: 150 cm) was admitted to our hospital and diagnosed as intermediate-risk B-cell ALL based on his morphology, immunophenotypic, and genetic features. According to the Chinese Children Cancer Group study ALL-2020 (CCCG-ALL-2020) protocol (referred to the 20-protocol; this study was registered at the Chinese Clinical Trial Registry [registration number: ChiCTR2000035264]), he began remission induction therapy (vincristine, daunorubicin, prednisone, and pegasparaginase [PEG-Asp]) on March 20, 2023. Unfortunately, the patient failed to achieve minimal residual disease (MRD)-negative complete remission (CR) on day 19 of induction, with a significant percentage of lymphoblasts (13.19%) in cerebrospinal fluid, thus reclassified as having a high-risk disease with central nervous system (CNS) involved. The patient experienced persistent coagulation dysfunction and hypofibrinogenemia following the application of PEG-Asp, but the baseline lipid and triglycerides (TG) levels were not assessed at that time. On day 30 of induction, the child suffered a 3-minute-long nighttime seizure, manifesting as disturbance of consciousness, diplopia, cyanosis of the lips, ptyalism, and tremors in the extremities, with no other neurological deficits. Then, these symptoms were promptly alleviated with an intramuscular injection of phenobarbital 0.1 g. An immediate brain computed tomography (CT) scan was performed, which could not rule out venous sinus thrombosis. Subsequent brain magnetic resonance venography (MRV) revealed frontal–parietal superior sagittal sinus, part of the cortical veins, and sigmoid sinus thrombosis. The patient continued with mannitol, phenobarbital, fibrinogen, and other symptomatic supportive treatments from days 30 to 33. Because of his stable condition and mild symptoms, anticoagulant therapy has not been initiated.

**Fig. 1:**
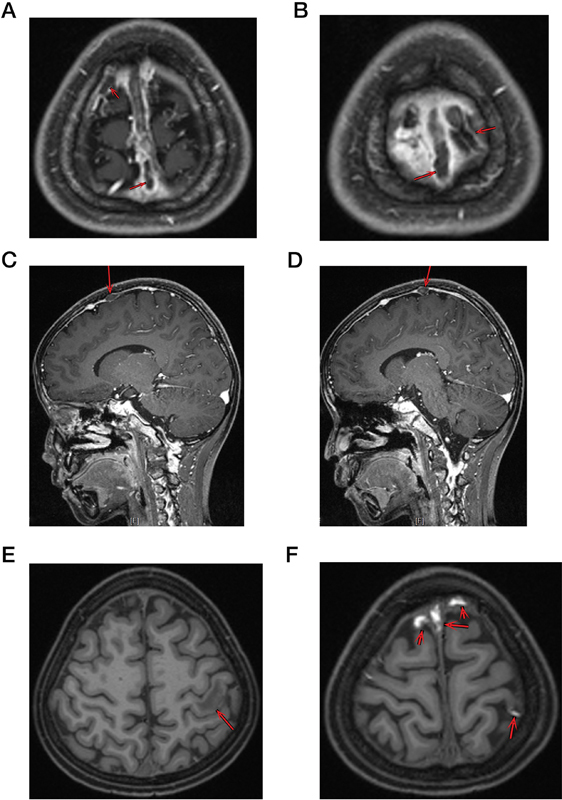
Radiographic data of first thromboses. ( **A** ) Right superior sagittal sinus thromboses in magnetic resonance venography (MRV) axial view. ( **B** ) Left superior sagittal sinus thromboses in MRV axial view. ( **C** ) Right superficial cutaneous vein thrombosis in MRV sagittal view. ( **D** ) Left superficial cutaneous vein thrombosis in MRV sagittal view. ( **E** ) Left postcentral gyrus ischemic focus in magnetic resonance imaging (MRI) analysis. ( **F** ) Superior sagittal sinus and superficial cutaneous vein thrombosis in MRI analysis. The red arrows shows where the thromboses are.

On day 33, the boy experienced another seizure lasting nearly 1 minute, characterized by unresponsiveness, tremors in the extremities, and extension of both lower limbs. Phenobarbital 0.1 g was administered immediately, alleviating these symptoms gradually. An urgent brain CT revealed intracranial thrombosis combined with hemorrhage, and MRV indicated that thrombosis in the superior sagittal sinus, cortical veins, and sinus confluence ( Fig. 1 ). He was transferred to the intensive care unit for further treatment. Immediate anticoagulation therapy was initiated with heparin at a dose of 0.2 mL every 12 hours (q12h). Additionally, intermittent transfusions of fibrinogen and fresh-frozen plasma were administered to rectify coagulation dysfunction. After 15 consecutive days of heparin administration, his neurological symptoms were relieved significantly, and MRV scans showed that the thrombus and hemorrhage had resolved with no other noticeable abnormalities. The patient was then transferred back to the general ward later.

He continued with the remaining remission induction and consolidation therapy based on the 20-protocol but failed to achieve MRD-negative CR throughout. From August 23, 2023, blinatumomab (17.5 µg/d; days 1–28) was administered, continuing with subsequent continuation therapy with high-dose methotrexate and triple intrathecal therapy every other week and daily mercaptopurine for four courses, and he eventually obtained MRD-negative CR. During the course of treatment, coagulation dysfunction and hypofibrinogenemia recurred but normalized after fibrinogen and plasma supplementation.

**Fig. 2:**
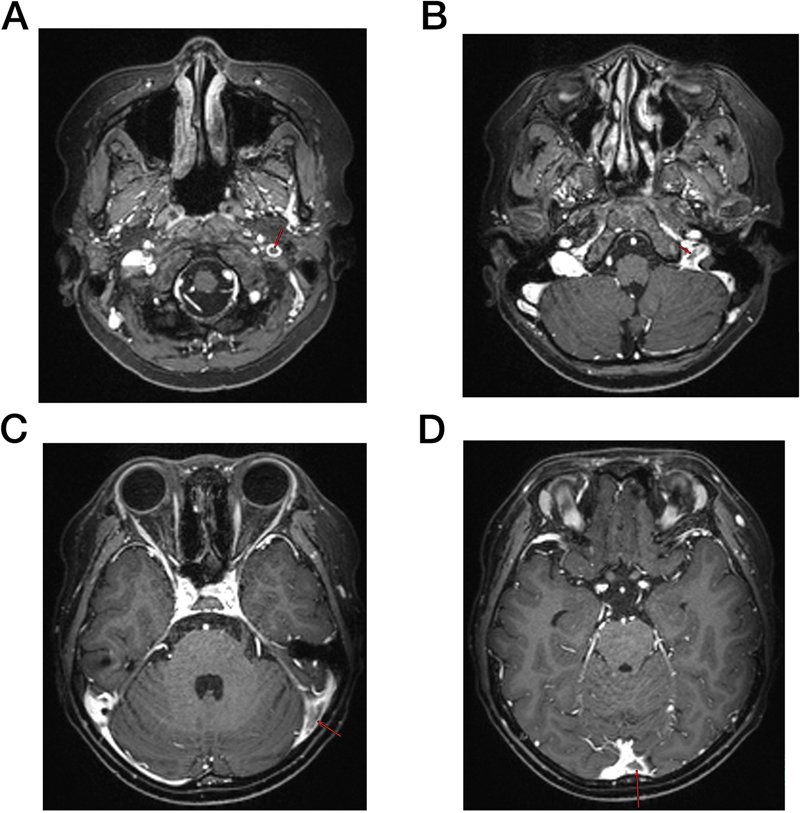
Radiographic data of second thromboses. ( **A** ) Thrombi in the left internal jugular vein in magnetic resonance venography (MRV) analysis. ( **B** ) Thrombi in the left sigmoid sinus in MRV analysis. ( **C** ) Thrombi in the left transverse sinus in MRV analysis. ( **D** ) Thrombi in sinus confluence in MRV analysis. The red arrows shows where the thromboses are.

**Fig. 3:**
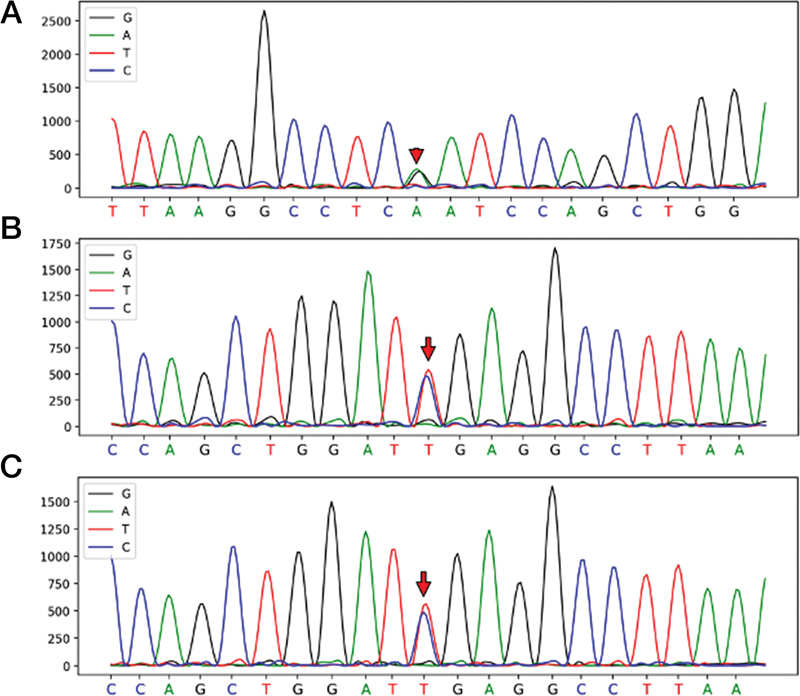
Gene mutation data. ( **A** , **B** ) The sequencing information of the patient. ( **C** ) The sequencing information of his father. The red arrows shows where the mutation are.

On December 5, 2023, the boy was readmitted due to wandering pain in the lumbar, back, and hip. Brain CT scan identified multiple thromboses in the left transverse and sigmoid sinuses, and magnetic resonance imaging and MRV revealed multiple thrombi in the left internal jugular vein, sigmoid sinus, transverse sinus, and sinus confluence ( Fig. 2 ). Immediate anticoagulation therapy with heparin 0.2 mL every 12 hours was administered. Additionally, a thrombophilia workup was performed to evaluate the acute thrombosis of unknown origin. The fibrinogen concentration was 0.98 g/L (normal range: 2–4 g/L), and lipid level was elevated, with TG 49.73 mmol/L, nearly 30 times above normal (range: 0–1.7 mmol/L), cholesterol 20.34 mmol/L, 5 times higher (range: 3.0–5.7 mmol/L), and low-density lipoprotein 0.63 mmol/L, 3 times higher (range: 1.89–4.21 mmol/L). Given the patients' young age and healthy lifestyle, genetic testing was performed to investigate potential thrombophilia-related and dyslipidemia-related genetic disorders as causes of abnormal hyperlipidemia and CVST. No thrombophilia-related gene mutation was detected, but a heterozygous mutation in lipoprotein lipase (LPL) gene (c.547G > A) was identified ( Fig. 3A, B ), which is associated with autosomal recessive inheritable LPL deficiency, indicating a high likelihood of hyperlipidemia. Considering the lack of guidelines for the use of fibrate–lipid-lowering drugs in children, atorvastatin 10 mg was initiated for lipid-lowering treatment. Despite this intervention, the TG level remained significantly elevated, approximately 15 times higher than normal. Consequently, atorvastatin was discontinued, and fenofibrate 0.1 g was introduced, resulting in a substantial decrease in TG level. Continuation therapy was commenced on December 21, 2023, and PEG-Asp was temporarily suspended due to persistently high TG levels. Given the genetic predisposition, relevant genetic and lipid examination was conducted on his parents as well, and his father was found to have the same LPL gene mutation and hyperlipidemia ( Fig. 3C ). To safeguard his health, we recommended the father immediately adopt initiate lipid-lowering treatment and adopt lifestyle modifications.

Later, he returned regularly for continuation therapy. Coagulation function and lipid profile were within normal range, but MRV results suggested left jugular vein, sigmoid sinus, transverse sinus, and sinus confluence thrombosis. Rivaroxaban 15 mg was administered orally for anticoagulation, along with fenofibrate for lipid-lowering treatment. Due to the potential negative impact of PEG-Asp on coagulation function and lipid levels, the long-acting PEG-Asp was replaced with short-acting Erwinia L-asparaginase (Erwinia-Asp). After the third term of Erwinia-Asp, lipid testing results revealed a recurrence of severe hypertriglyceridemia (HTG), with TG levels of nearly 13.09 mmol/L. Consequently, Asp was promptly discontinued.

The patient is now in long-term lipid-lowering therapy with fenofibrate, and intermittent TG levels are within normal range. Scheduled bone marrow assessments have been carried out to monitor the remission status of the primary disease status, and he remains in an MRD-negative CR state currently.

## Discussion

A previous meta-analysis of 17 prospective studies calculated the thrombosis rate in pediatric ALL patients to be 5.2%, with most thromboses occurring during induction therapy. 5 6 7 The overall rate of cerebral thrombosis was found to be 2.9%. 8 During the course of ALL treatment, hyperlipidemia tends to occur at two main time points: weeks 4 to 6 and months 5 to 7 of induction, typically following the administration of L-Asp. In the case of the study, the patient developed hyperlipidemia and CVST during L-Asp treatment, with a dyslipidemia-related but no thrombophilia-related genetic mutation detected. neurological symptoms were managed with short-term anticoagulant therapy and long-term lipid-lowering therapy.

Hyperlipidemia is associated with thrombosis and other severe complications, but no standard recommendations are provided for screening or monitoring of hyperlipidemia in the treatment of L-Asp. 6 9 10 11 12 13 14 While the elevated TG levels in pediatric ALL patients are generally transient and rarely necessitate the suspension of therapy, significant increases in TG levels are thought to be attributed partially to genetic predisposition, 15 as evidenced by the child in this case who exhibited abnormal TG and LPL gene mutation during L-Asp treatment. LPL gene mutation could affect the enzyme responsible for breaking down TG in chylomicrons and very low-density lipoprotein. Deficiency in lipoprotein lipase could lead to HTG. Accordingly, it is prudent to monitor baseline lipid profile and dyslipidemia-related gene testing at the start of L-Asp treatment and continue periodic monitoring throughout the course of ALL therapy, a limitation we acknowledge in this case. Further studies are essential to identify children who might potentially benefit from increased monitoring of TG levels and relevant gene testing.

CVST often occurs during induction therapy, particularly after L-Asp infusion, and its occurrence appears to be unrelated to CNS leukemia. 6 The mechanism by which L-Asp induces thrombosis and CVST is complex, and L-asp is believed to disrupt the physiologic balance between the hemostatic and anticoagulation pathways, alongside activating platelets and endothelial cells. Risk factors for venous thromboembolism and CVST included TG > 500 mg/dL, thrombophilia, elevated coagulation factors, high-risk ALL, and chemotherapeutic agents, of which HTG and thrombophilia proved to be independent predictors. 15 16 17 Hereditary factors were reported to increase the risk of thrombosis by 8.5 times in pediatric patients treated with L-asp. 17 In the clinical case above, the patient was diagnosed with genetic HTG and concomitant CVST, but no mutations related to thrombophilia were detected. We speculate that dyslipidemia-related gene mutation contributed to HTG, thereby increasing the risk of CVST. This rationale underpins our decision to conduct dyslipidemia- and thrombophilia-related gene testing for the child, as we believe relevant gene screening throughout the treatment to be valuable. Based on these findings, we suspected that it is crucial to conduct not only thrombophilia-related gene testing but also dyslipidemia-related gene testing when administering drugs with a high risk of intriguing hyperlipidemia and CVST.

There are currently no standardized guidelines for the management of CVST during ALL treatment. Anticoagulation involving heparin and low molecular weight heparin remains the primary therapeutic approach for CVST, with mechanical thrombectomy and thrombolysis indicated when necessary. While the safety and efficacy of direct oral anticoagulants (DOACs) have been confirmed in adults, there has been no clinical experience in children. 18 Previous studies have recommended prophylactic anticoagulation for patients at high risk of CVST and emphasized immediate initiation of anticoagulation for children and adolescents with CVST, irrespective of the presence of hemorrhage. 13 19 Therefore, we recommend that pediatric ALL patients who experienced CSVT receive anticoagulation therapy as early as possible. As to prophylactic anticoagulation and the application of DOACs in pediatric ALL patients, further research is required to ascertain its necessity.

Debate continues regarding the safety of L-Asp reexposure, dose adjustment, or permanent discontinuation after CVST. 19 In this case, we restarted L-Asp with short-acting products after the child recovered from neurological symptoms, but permanently discontinued L-Asp due to recurrent HTG and CVST. Decisions about rechallenging with L-Asp who experienced L-Asp-induced HTG and CVST must be made on personalized analysis. Consequently, we emphasize the urgent need for developing management strategies and standard guidelines for patients who developed HTG and CVST during ALL therapy.

## Conclusion

In the treatment of childhood ALL, L-Asp is a critical component, playing a crucial role in both the induction and intensification phase, but L-Asp always contributes to several unique and potentially lethal toxicities. Genetic screening for thrombophilia-related mutation before L-Asp treatment, as well as lipid level monitoring during treatment course, and dyslipidemia-related gene mutation when necessary, may be of clinical significance for clinical decisions. Future research should focus not only on the guidance of managing the complication of L-Asp but also on exploring methods to reduce the side effects of L-Asp.

**Table 1:** The coagulation function and lipids level at key time points

Time	Events	PT	APTT	TT	Fibrinogen	FDP	D-dimer	TG	CHO	HDL	LDL
April 18, 2023	First CVST (first seizure)	13.4	28.1	22.5	0.63	17	4.04	–	–	–	–
April 18, 2023	–	12.7	29.5	20.3	0.98	15.9	4.26	–	–	–	–
April 19, 2023	–	13.2	26.6	20.7	0.88	17.9	4.81	–	–	–	–
April 20, 2023	–	13	27.7	20.5	0.86	13.5	3.68	–	–	–	–
April 21, 2023	First CVST (second seizure)	12.7	29.6	23.2	0.84	10.3	3.58	–	–	–	–
April 21, 2023	–	12.3	28.2	22.6	1.03	11.5	3.47	–	–	–	–
April 22, 2023	–	12.9	31.4	24.9	1.43	12.7	4.00	–	–	–	–
He continued with the remaining remission induction and consolidation therapy, and coagulation dysfunction and hypofibrinogenemia recurred but normalized after fibrinogen and plasma supplementation											
December 7, 2023	Second CVST	11.7	26.7	20.1	2	<2	0.88	–	–	–	–
December 8, 2023	–	12.5	32.8	32.2	1.75	<2	0.65				
December 9, 2023	–	13.1	32.2	24.7	1.74	<2	0.55	49.73	20.34	4.04	0.63
December 12, 2023	–	13.2	29.9	18.7	1.87	<2	0.56	21.49	20.66	4.3	1.03
December 14, 2023	–	13.2	33	20	1.59	<2	0.42	–	–	–	–

Abbreviations: APTT, activated partial thromboplastin time; CHO, cholesterol; CVST, cerebral venous sinus thrombosis; FDP, fibrinogen degradation products; HDL, high-density lipoprotein; LDL, low-density lipoprotein; PT, prothrombin time; TG, triglycerides; TT, thrombin time.
